# Impact of 4.0% chlorhexidine cleansing of the umbilical cord on mortality and omphalitis among newborns of Sylhet, Bangladesh: design of a community-based cluster randomized trial

**DOI:** 10.1186/1471-2431-9-67

**Published:** 2009-10-21

**Authors:** Luke C Mullany, Shams El Arifeen, Peter J Winch, Rasheduzzaman Shah, Ishtiaq Mannan, Syed M Rahman, Mohammad R Rahman, Gary L Darmstadt, Saifuddin Ahmed, Mathuram Santosham, Robert E Black, Abdullah H Baqui

**Affiliations:** 1 Department of International Health, Johns Hopkins Bloomberg School of Public Health, Baltimore, MD, USA; 2 International Centre for Diarrhoeal Disease Research, Bangladesh (ICDDR,B), Dhaka, Bangladesh; 3 Department of Population, Family, and Reproductive Health, Johns Hopkins Bloomberg School of Public Health, Baltimore, MD, USA

## Abstract

**Background:**

The World Health Organization recommends dry cord care for newborns but this recommendation may not be optimal in low resource settings where most births take place in an unclean environment and infections account for up to half of neonatal deaths. A previous trial in Nepal indicated that umbilical cord cleansing with 4.0% chlorhexidine could substantially reduce mortality and omphalitis risk, but policy changes await additional community-based data.

**Methods:**

The Projahnmo Chlorhexidine study was a three-year, cluster-randomized, community-based trial to assess the impact of three cord care regimens on neonatal mortality and omphalitis. Women were recruited mid-pregnancy, received a basic package of maternal and neonatal health promotion messages, and were followed to pregnancy outcome. Newborns were visited at home by local village-based workers whose areas were randomized to either 1) single- or 2) 7-day cord cleansing with 4.0% chlorhexidine, or 3) promotion of dry cord care as recommended by WHO. All mothers received basic messages regarding hand-washing, clean cord cutting, and avoidance of harmful home-base applications to the cord. Death within 28 days and omphalitis were the primary outcomes; these were monitored directly through home visits by community health workers on days 1, 3, 6, 9, 15, and 28 after birth.

**Discussion:**

Due to report in early 2010, the Projahnmo Chlorhexidine Study examines the impact of multiple or single chlorhexidine cleansing of the cord on neonatal mortality and omphalitis among newborns of rural Sylhet District, Bangladesh. The results of this trial will be interpreted in conjunction with a similarly designed trial previously conducted in Nepal, and will have implications for policy guidelines for optimal cord care of newborns in low resource settings in Asia.

**Trial Registration:**

ClinicalTrials.gov (NCT00434408)

## Background

Of the annual 3.7 million global neonatal deaths, almost all (99%) occur in developing countries, and one-third to one half are attributed to infections[1] such as sepsis, meningitis, pneumonia, omphalitis, tetanus, and diarrhea. In settings where home delivery is common and attendance by skilled personnel is low, many babies are born in unhygienic conditions and infections of the umbilical cord stump are common [2,3]. Exposure of the freshly cut umbilical cord stump to pathogens on the cutting instrument, on the hands of caretakers, or elsewhere in the environment leads to local cord infections (omphalitis) that may progress to systemic infection and death [2-4]. Alternatively, during the period immediately following birth, while the vessels of the umbilical cord remain patent, direct exposure to the bloodstream via the cord may lead to systemic infection without any visible signs of local cord infection [5].

The World Health Organization (WHO) currently recommends keeping the cord clean and dry. The guidelines do not include a universal recommendation for application of any topical antiseptic, although their potential role is acknowledged for settings where harmful practices are common [2]. Practical and effective implementation of this guidance, however, is challenging in both hospital and community-based low resource settings and is often less than optimal. Recently, there has been increased interest in both the overall role of exposure of the cord stump to invasive pathogens and the potential benefit of topical cord cleansing with chlorhexidine[6], a safe and effective broad-spectrum antiseptic[7].

A large community-based trial[8] in rural southern Nepal conducted between 2002 and 2006 randomized babies within clusters to receive one of three cord care regimens: (1) 4.0% chlorhexidine cleansing for 7 of the first 10 days after birth or (2) soap and water cleansing for 7 of the first 10 days after birth, or (3) dry cord care. Mothers of all infants received educational messages about hygienic cord care (e.g., hand-washing, clean cutting, and avoidance of harmful topical applications). Depending on the severity of infection, omphalitis incidence was reduced by 32-75% among infants receiving chlorhexidine compared to those in the dry cord care group. Overall, mortality among enrolled infants was 24% lower in the chlorhexidine group compared to dry cord care. About two-thirds of the infants were reached within 24 hours of birth and evidence of a protective benefit of chlorhexidine cleansing among this subset was increased. Chlorhexidine cleansing reduced severe infection by 87% and mortality by 34% among those enrolled within 24 hours, while no difference between the groups was observed when cord cleansing was initiated after 24 hours[8].

An expert consultative meeting convened in Washington, DC shortly after completion of the Nepal trial concluded that, prior to any changes in policy, at least one replication trial in South Asia would be necessary to expand the evidence base for chlorhexidine cleansing of the cord[9]. Two specific questions were noted. First, would a similar beneficial effect of multiple cleansings of the cord through the first week of life be observed in a separate but similar population? Second, can a simpler regimen of cord cleansing only once and as soon as possible after birth, provide a similar protective benefit against omphalitis and mortality? Answering this second question is necessary as the intensive intervention (cleansing on 7 of first 10 days of life) followed in the Nepal efficacy trial might not be easily implemented in most programmatic settings, given associated logistical and financial barriers for outreach health workers. Furthermore, even if the intervention was delivered by caretakers themselves, compliance with the promoted instructions might be increased if the more simple recommendation of one-time cleansing as soon as possible after birth could be given. Moreover, a single application of antiseptic to the cord could more readily be incorporated into clean birth kits.

Thus, in addition to replicating the question of efficacy of multiple (i.e. daily for 7 days) cord cleansing with chlorhexidine (4%, as used in Nepal), it was deemed essential to also investigate the potential efficacy of a single application as soon as possible after birth. The second major research activity for the Projahnmo study site in rural Sylhet District of north eastern Bangladesh was designed to answer these questions and provide evidence to inform global guidelines for optimal umbilical cord care of newborns. In this manuscript, we discuss in detail the design and implementation of this community-based cluster randomized trial, including study site, randomization and intervention delivery procedures, follow-up data collection, and analysis plans.

## Methods/Design

### Trial Design and Preparation

#### Aim

This community-based trial in Sylhet, Bangladesh was designed to assess the impact of chlorhexidine cleansing on neonatal mortality and omphalitis. Specifically, these outcomes were compared among babies randomly assigned within clusters to one of three cord care regimens: **1) Multiple Chlorhexidine Cleansing Group**: Neonates received cleansing of the cord with 4.0% chlorhexidine as soon as possible after birth and on subsequent days until age 7 days; **2) Single Chlorhexidine Cleansing Group**: Neonates received a single cleansing of the cord with 4.0% chlorhexidine as soon as possible after birth. **3) Dry Cord Care Group**: Neonates in this group received no specific umbilical cord care ("dry cord care", as recommended by WHO) beyond the basic messages related to clean cord cutting and avoiding home-based applications to the cord, which were directed to all groups. Mothers and newborns in all three groups also received a basic community-based package of essential newborn care (described below).

#### Study Site

This study was conducted in three sub-districts (*upazillas*) of Sylhet District (Zakiganj, Khanaighat, Beanibazar) of north eastern Bangladesh. This is the research site of Projahnmo, a collaboration of the Johns Hopkins Bloomberg School of Public Health, the International Centre for Diarrhoeal Disease Research, Bangladesh (ICDDR,B), the Ministry of Health of the Government of Bangladesh, and Shimantik , a non-governmental organization. In the first Projahnmo study, details of which have been previously published[10], 24 unions (a government-defined administrative sub-unit of the *upazilla*, of approximately 20,000 population) in this region were randomized to one of three arms. Two arms received a newborn care intervention, delivered either through community-mobilization activities or a more intensive home-care approach (pregnancy and postnatal visits), while the third arm received currently available care, and served as the comparison arm. In the home-care arm, community-based health workers (CHWs) were additionally trained to assess newborns for severe illnesses and refer or treat sick newborns. In that trial, neonatal mortality was reduced by 34% in the home-care arm relative to the comparison arm[10]. This study site and the home-care intervention including essential newborn care promotion and follow-up assessments of newborns formed the basis of the infrastructure and service provision upon which the Projahnmo Chlorhexidine Study was implemented.

#### Study Population and Surveillance Infrastructure

**Figure 1:**
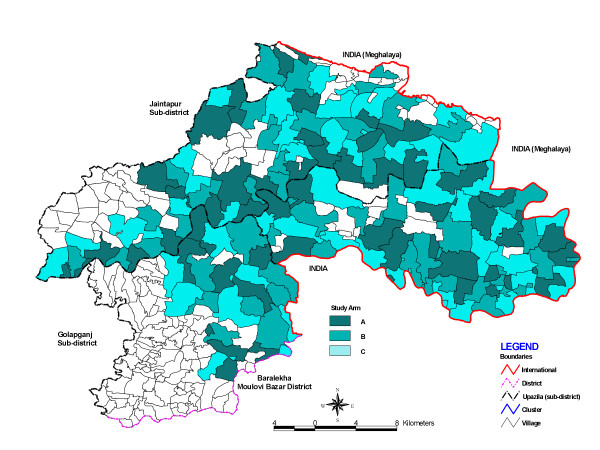
**Map of Sylhet District, Bangladesh and unions participating in the Projahnmo Chlorhexidine Study**.

Six lower mortality unions of the original 24 Projahnmo unions were excluded, and 4 additional higher-mortality unions from Khanaighat were added, leaving 22 total unions for participation in the study (Figure 1 - Map). The study population for the Projahnmo Chlorhexidine Study was defined as all live-born infants delivered in these 22 unions, and the total population was approximately 546,000 with an expected annual birth cohort of 12,000 to 13,000 babies. Prior to the initiation of trial activities, the project team undertook a community mapping and household enumeration exercise to delineate the CHW surveillance areas that served as the clusters for this trial. CHW surveillance areas were pre-established for 6 unions (33 CHW areas) that had participated in the home-care arm of the previous trial. The remaining 18 unions had either previously participated in the community-care arm (5 unions) or comparison area (7 unions), or were new expanded areas (4 unions). In these 18 unions, delineation of new CHW areas was required, and the mapping/enumeration process resulted in 32, 40, and 28 CHW areas in the former community care, comparison, and the new expanded areas, respectively. Over the 133 total study clusters, the mean population size and standard deviation was 4,108 and 633, respectively (range: 2,071 - 5,598).

Each defined CHW area (cluster) was further divided based on population size into 4-5 village-based sub-sections of approximately 1000 population (~200 households) each. Within each cluster, a locally resident female CHW was recruited and trained in all aspects of the study. Similarly, within each of the cluster sub-sections (n = 590), a single female village-based health worker (VHW) was identified to assist directly with delivery of the cord care intervention.

#### Randomization

The unit of randomization was the CHW area (n = 133); 105 of these had participated in one of the three arms of the previous Projahnmo project (home-based care: n = 33; community-care: n = 32 clusters; comparison arm: n = 40 clusters). As there were differences in neonatal risk within these three strata during the previous study, the allocation of clusters in the current study was done separately within each of these strata to facilitate balance in baseline neonatal mortality risk. A fourth stratum was defined for the remaining 28 clusters from the expanded area (i.e. not participating in the previous project). A random allocation sequence for each stratum was done using a computer-generated procedure (STATA version 9.2, StataCorp., College Station, TX, USA). Within strata, clusters were randomly allocated to multiple cleansing, single cleansing, or dry cord care arms using the procedure with a constant block size of three. The distribution of clusters over the three allocation groups and previous project participation status, along with numbers of VHWs is shown in Table 1. Workers and participants in the study were not masked to group allocation.

**Table 1:** Characteristics of the clusters participating in the Projahnmo Chlorhexidine Study and distribution across study arm and previous project status

	**Multiple Cleansing**	**Single Cleansing**	**Dry Cord Care Arm**	**Overall**
**Total Number**	45	44	44	133

**Previous Projahnmo Status**				

**- Home Care Arm**	11	11	11	33

**- Community Care Arm**	11	10	11	32

**- Comparison Arm**	14	13	13	40

**- Expanded Areas**	9	10	9	28

**Total Population Size**	188,479	177,110	180,880	546,469

**Mean Population Size**	4,188	4,025	4,110	4,108

**Number of VHW areas**	202	195	193	590

#### Preparation of Chlorhexidine

At regular intervals, 20% chlorhexidine digluconate was purchased in bulk from ACI Limited, Bangladesh and transferred to Sylhet in 5 litre containers. The source manufacturer for ACI was Vipor Chemicals PVT. LTD  based in Baroda, India. A Projahnmo worker prepared the diluted 4.0% chlorhexidine solution for use in the trial, by mixing distilled water (645 ml/litre) with the stock solution (355 ml/litre) to the appropriate concentration (7.1% of the total digluconate salt); this was identical to the dilution process in the previous Nepal trial. The diluted solution was individually packaged into 70 ml and 10 ml opaque bottles for distribution to VHWs in the multiple and single cleansing groups, respectively. For each batch of prepared solutions, 5 bottles were randomly selected, transferred to a collaborating pharmaceutical laboratory at Eskayef Bangladesh Limited, and analyzed for final CHX concentration.

### Trial Implementation

#### Enrolment of Pregnant Women and Basic Intervention Package

Identification and recruitment of pregnant women was based on regular monitoring by CHWs of printed registers containing the names and locations all married women of reproductive age (MWRA) within their individual target areas. Workers updated the list of MWRA and ascertained the pregnancy status of all women on the register by conducting an informal survey of each household in their area once every 8 weeks. The study was explained, participation offered, and informed consent provided to each newly pregnant women identified through this monitoring process. Those agreeing to participate provided data including age, parity, date of last menstrual period, occupation, literacy, abbreviated birth history, and socio-economic information about the household.

Participating women were provided with a basic package of maternal and newborn health promotion messages and interventions, delivered at two home visits during pregnancy. The first of these sessions was conducted at the same time as the recruitment visit at about 3-4 months of pregnancy; the second occurred at approximately the 8^th ^month of pregnancy. At each session, messages and counselling were provided regarding pregnancy care (antenatal care-seeking, tetanus toxoid immunization, iron/folic acid supplementation, and recognition of danger signs during pregnancy and at the time of delivery), identification and selection of a birth attendant, clean and hygienic delivery, early and exclusive breastfeeding, thermal care of the newborn, and postnatal danger signs and care-seeking. All families were provided a clean birthing kit and the use of items in the kit was explained through demonstration at these pregnancy visits.

Sessions during pregnancy provided opportunities to establish the process for notification of project workers regarding pregnancy outcomes, and to reinforce the importance of accomplishing this as rapidly as possible. By accompanying the CHWs on their two pregnancy home visits, the VHWs were able to orient the family members on the process of sending a message to the VHW via a family member or another individual when the pregnant women began labor. Compliance with this notification system was strengthened by explaining that rapid notification would enable faster assessment of the health of the baby by the CHW.

#### Chlorhexidine Intervention

After being notified of an ongoing labor or recent delivery, the local VHW travelled to the home of the newborn to deliver the assigned intervention. Depending on the cord care regimen randomly allocated to that location (i.e. cluster), the VHW either provided the standard set of dry cord care promotional messages (dry cord care group) or applied the initial cleansing of the cord (single and multiple cleansing groups). VHWs in **all **groups visited the home of the newborn daily through the first week of life (i.e. 7 days) and basic messages about cord hygiene were promoted across all the groups at these visits. In addition, the VHW provided the chlorhexidine cleansing intervention at each visit in the multiple-cleansing arm, and at the first visit only in the single-cleansing arm. Each individual VHW consistently followed only one set of procedures throughout the trial period, as the unit of randomization was the area (cluster) of the VHW's supervising CHW. The VHW recorded the date and approximate time of visits, the vital status of the baby (alive/dead), and the status of the intervention for that day (provided, refused) on a pictorial form.

For newborns receiving chlorhexidine interventions (i.e. arms 1 and 2), the basic cleansing procedure followed by the VHW on day 1 was consistent with how the intervention was delivered in the previous community-based trial in rural Nepal[8]. First, the VHW washed her hands with soap and water and allowed them to air dry. If the baby was wrapped or clothed, the VHW removed an amount of covering necessary to expose the cord stump area. She moistened a cotton swab by placing it on the mouth of the chlorhexidine bottle (10 ml or 70 ml bottle, depending on study allocation) and inverting the bottle. The worker then gently dabbed the abdominal skin around the base and cord stump thoroughly, ensuring that the entire area was covered with solution. A second cotton swab was similarly moistened and used to cleanse the tip of the cord (if not yet separated) or the middle of the cord stump (if separation had occurred). After cleansing the cord, the VHW wrapped the baby warmly and placed the baby in the arms of the mother or other family member. Emphasis was placed on thoroughly completing the cleansing procedure as quickly as possibly, followed by prompt wrapping of the baby.

#### Follow-up Visits

In all clusters, after conducting the first visit after birth, the VHW notified their supervising CHW through direct communication, mobile phone, or secondary messenger. The supervising CHW then visited the home to initiate the neonatal follow-up and assessment schedule. At the first follow-up visit by the CHW (day 1), basic information regarding the characteristics of labor and delivery and morbidity prior to, during, and after delivery was recorded. Newborn-specific data collected at this visit included date and time of birth, sex, weight of the infant, and care given during and immediately after birth (bathing, massage, breastfeeding practices). At this first visit and subsequent assessment visits (days 3, 6, 9, 15) the CHW assessed the status of the newborn through a combination of direct observation (vital status, axillary temperature, chest indrawing, umbilical and skin infection, jaundice, lethargy, respiratory rate, etc) and questions directed to the mother regarding signs of morbidity of the infant and care-seeking since the prior visit (or since birth).

A particular focus was placed on assessing signs of umbilical cord infection. Previous community-based work in Nepal[11] and Tanzania[12] utilized a standard set of fifty digital images of umbilical cords with varying degrees of inflammation to assess intra- and inter-worker agreement in recognition of signs and to estimate sensitivity and specificity of workers compared to a physician gold standard ranking of the images. Following this model, CHWs were trained to recognize and classify the severity of three signs of infection: redness, pus, and swelling, and their skills were evaluated through repeated (quarterly) assessment of these images. Results were used to identify those CHWs needing more focused one-on-one training.

A sign-based algorithm [13] was followed by the CHWs to identify sick newborns and refer them for care. In the 33 clusters from the home-care arm of the previous research study, CHWs also had the capacity to treat newborns at home with injectable antibiotics, following procedures that have been previously described[14]; these clusters were balanced across the three cord care regimens (11 clusters in each arm of this study, Table 1). At a final visit conducted between 28 and 35 days after birth, the CHW recorded the vital status of the baby.

Any neonatal death occurring during the period of follow-up was recorded; at regular intervals a database-generated list of recent deaths were distributed to supervisory staff responsible fro conducting an in-depth verbal autopsy. An automated algorithm utilized sign-based cause-of-death definitions to assign multiple and/or single cause of death to each infant, using both hierarchical and non-hierarchical assignment [15].

### Outcomes, Sample Size, and Analysis

#### Eligibility and Enrolment Criteria

**Figure 2:**
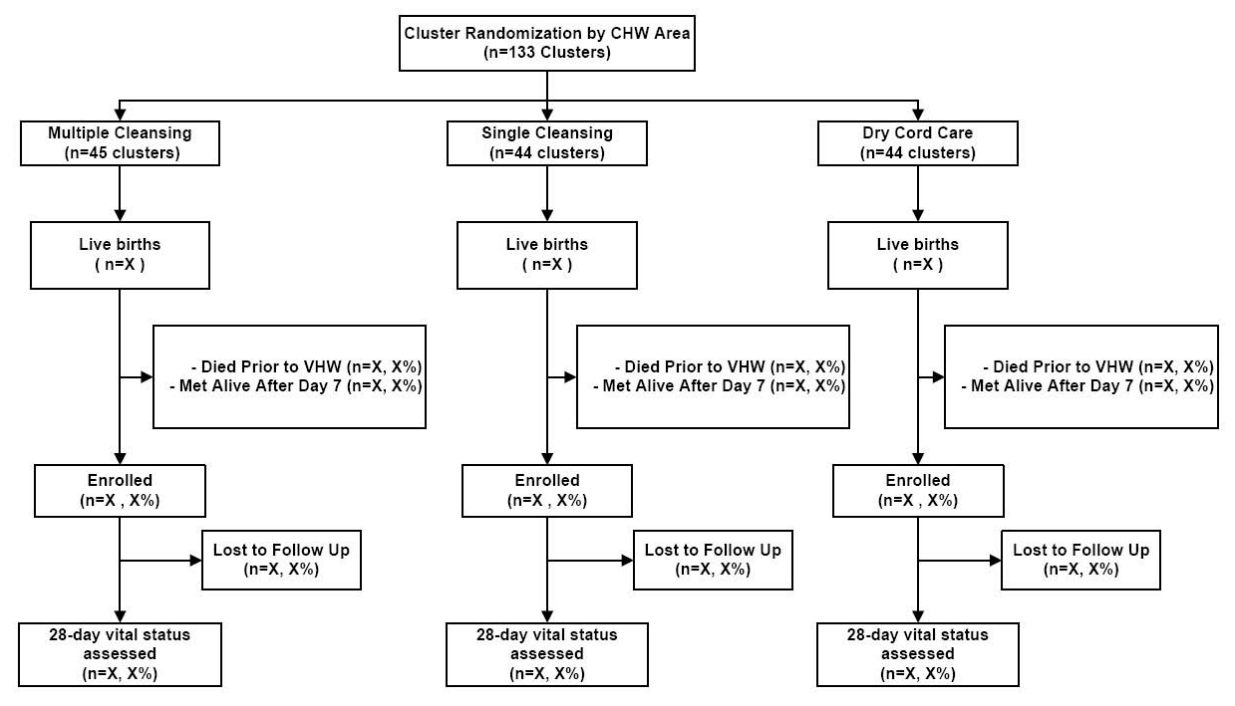
**Trial Design Flowchart**.

All live born babies in the study area were eligible for the study (Figure 2). Babies were enrolled if 1) they were alive at the time of the VHWs first home visit and 2) this first visit occurred within 7 days after birth. All enrolled babies will be included in the primary intent-to-treat analysis, regardless of the status of receipt of the allocated intervention. Some pregnant women delivered in a location other than the location where the initial pregnancy was identified and data were collected. For example, in this region approximately 15% of women move late in pregnancy in order to deliver in their maternal home and 10-12% deliver in a health facility. In this trial, all neonates were assigned the treatment code corresponding to the cluster in which they were born, regardless of whether or not the birth cluster matched the one in which the mother was first enrolled. Infants born in a facility were enrolled in the cluster in which they were first met after returning from the facility. That is, infants born alive in a facility were considered eligible, and were included in the primary analysis if enrolled (i.e. if they were met at home by a project worker during the first seven days of life).

#### Outcome Measures

The two primary outcomes of this trial were neonatal mortality and omphalitis among live born enrolled infants. Neonatal mortality was defined as death within the first 28 days after birth and the sample size calculation (see ***Sample Size***) was based on detection of mortality difference among enrolled infants. Omphalitis was defined under various sign-based algorithms representing mild, moderate, and severe omphalitis[11,12]. Other *a priori *planned outcome analyses include 1) an assessment of the impact of the intervention on these outcomes among the entire sample of live born infants, including those that died before they had an opportunity to be enrolled in the trial, 2) analyses stratified by birth weight status (<2500 grams vs. > 2500 grams), gestational age (<37 weeks vs. >37 weeks), timing of initiation of intervention, and 3) analyses conditioned on survival to various time points (i.e. 24 hours, 48 hours, etc).

#### Sample size

**Figure 3:**
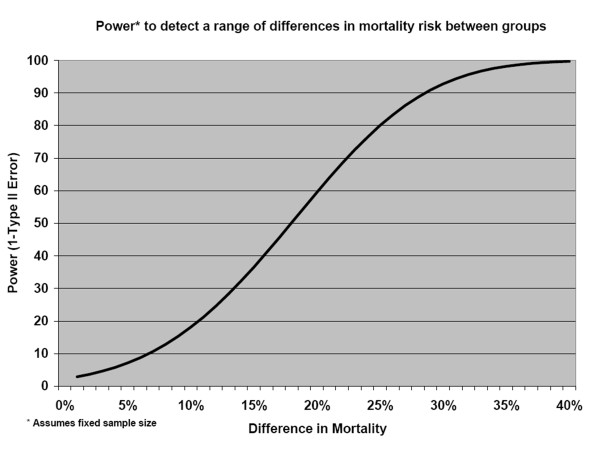
**Power to detect a range of differences in mortality risk between group**.

We hypothesized that single- or multiple-umbilical cord cleansing with 4.0% chlorhexidine solution would result in a 20-30% reduction in neonatal mortality among enrolled infants, based on reductions observed in the previous Nepal trial. Sample size calculations were based on 20% Type II error (80% power), 5% Type I error, and10% loss to follow-up, and the presumed design effect (1.2) was estimated from mean cluster size and coefficient of variation from the previous Projahnmo activities. The expected neonatal mortality rate (NMR) in the dry cord care group was estimated at 36/1000, which accounted for a 30% reduction in NMR expected after implementing the basic package across all 133 clusters. As not all infants were expected to be met immediately at birth, sample size calculations were based on assuming that a proportion of NMR would occur prior to enrolment. Varying this proportion and the overall desired detectable reduction in mortality among enrolled infants enabled estimation of required sample sizes (Table 2). We ultimately assumed that 20% of neonatal deaths would occur prior to enrolment, requiring approximately 28,797 newborns (9,600 in each group) to detect a difference of 25% in mortality among enrolled infants. A power curve displaying the power to detect a range of differences under this fixed sample size is shown in Figure 3. At a rate of 12,500 births per year, we expected to fully enrol the required number within 2.3 years. Due to the size of the study area and the large number of clusters, the trial initiation was phased-in across the 133 clusters between June and December of 2007; the staggered implementation was balanced on treatment groups.

**Table 2:** Total required number of newborns required for a range of detectable differences in mortality, by proportion of NMR occurring prior to enrolment

	**Detectable Difference in Mortality**		
	
**Proportion of neonatal mortality occurring prior to enrolment**	**20%**	**25%**	**30%**
**10%**	40643	25511	17367

**20%**	45883	**28797**	19602

**30%**	52620	33022	22474

#### Data Management and Analysis

CHWs collected data using maternal-level forms during their routine area monitoring and the two pregnancy visits, and infant-level forms during their follow-up visits to the newborns. The VHW-collected pictorial information on intervention visits in the first 7 days of life (date, time, visit status) was confirmed by CHWs through discussion with family members and then transferred to the infant-level forms maintained by the CHWs. All forms were checked for completeness daily by CHWs and at fortnightly meetings with supervisory staff, and then transferred to Dhaka for entry into a customized Microsoft SQL Server database. Entry programs included standard range and validation checks. Selected process-related data variables were entered at the field level to enable rapid feedback on essential implementation variables (e.g. enrolment, coverage and timing of home visits, etc.) to the field level supervisory staff.

For the final (and interim) analyses descriptive statistics on enrolment rates, timing of intervention delivery, frequency and coverage of home visits, receipt of components of the basic package, reported care practices, and health and demographic characteristics of newborn, mother, and households will be examined for balance across the groups. The primary impact analyses will follow an intent-to-treat approach among all enrolled infants, and will focus on comparing the mortality experience of newborns across the three cord care regimen groups. The mortality rate per 1000 live births will be estimated for all groups, and risk of death in each of the intervention groups (multiple and single cleansing) relative to the dry cord care group will be calculated. Standard errors will be adjusted for the clustered allocation using the generalized estimating equation approach[16], and 95% confidence intervals will be estimated. If necessary, adjusted models will be constructed to account for any imbalance between the groups.

Individual signs of cord infection and omphalitis under three sign-based algorithms (mild, moderate, severe) will be compared between groups using similar regression models with adjustment for both clustered design and multiple measures per baby. Individual visit level cord assessment data will be collapsed over the neonatal period with binary indicators created for each of the sign-based algorithms to estimate cumulative incidence of cord infection and risk of omphalitis relative to the comparison dry cord group.

#### Data Safety and Monitoring Board and Interim Analyses

An independent Data Safety Monitoring Board (DSMB) consisting of national and regional experts was established. Interim analyses were conducted after 31.3% (March 2008) and 69.8% (January 2009) of the expected sample size was recruited. These data were presented to the DSMB in both electronic reports and face-to-face meetings in Dhaka, Bangladesh. Stopping guidelines for efficacy were agreed upon by the DSMB members and the investigating team prior to the trial, using Lan-DeMets boundaries and nominal p-values for group sequential analyses estimated using O'Brien/Fleming spending function[17,18]. At each of the meetings, the DSMB recommended continuation of the project as originally planned.

#### Approvals

The study protocol was approved by the Institutional Review Board of the Johns Hopkins Bloomberg School of Public Health and the Ethical Review Committee of the International Centre for Diarrheal Disease Research, Bangladesh (ICDDR,B). The protocol has been reviewed and re-approved on an annual basis since 2007.

## Discussion

Due to report in early 2010, the Projahnmo Chlorhexidine Study will provide an evaluation of the impact of multiple- or single cord cleansing with 4.0% chlorhexidine relative to the current WHO recommended dry cord care on neonatal mortality and omphalitis. The results from this trial will expand the evidence base for the use of topical chlorhexidine as a low-cost, simple-to-deliver intervention, and it's potential to reduce neonatal mortality and morbidity in low resource settings. Furthermore, it will provide new information on one-time cleansing of the umbilical cord and its potential effect relative to multiple cleansing. Since the chlorhexidine intervention was implemented in the context of a basic package of maternal and newborn care, this study will provide data on the impact of chlorhexidine cleansing which is additional to the effect of the basic package of care which is being scaled up in Bangladesh.

Rural Sylhet District of Bangladesh is an appropriate location for examining the impact of chlorhexidine interventions, with strong potential for generalizing results to the wider South Asian region. The rate of home births, overall neonatal mortality rates, and the proportion of deaths attributed to infection in this region are high and comparable to large parts of Bangladesh, India, Nepal and Pakistan. Access to care and care-seeking by households is low and identification of interventions that can improve survival and be delivered by community-based workers or caretakers in the household is urgently needed. The existing Projahnmo Project provided a highly capable and well-developed infrastructure for conducting a population-based community trial. Most importantly, Projahnmo represents a strong partnership that includes the Government of Bangladesh, local and international research institutes and NGOs. This broad-based partnership will increase the likelihood that if the intervention is found successful, it will be incorporated into national policy recommendations and scaled up within Bangladesh.

The data from this trial will be interpreted in conjunction with the results from the previous Nepal trial and will further inform ongoing discussions regarding current WHO recommendation for dry cord care. Further community-based trials are being planned for settings in sub-Saharan Africa, and lessons learned from the design and implementation of this trial might provide guidance to the conduct of those trials. If the intervention is ultimately proven successful, chlorhexidine cleansing of the cord can be rapidly incorporated into existing neonatal care promotion programs and contribute to global efforts to improve newborn survival.

## List of Abbreviations Used

CHW: Community Health Worker; NMR: Neonatal Mortality Rate; Projahnmo: Project to Advance the Health of Newborns and Mothers; VHW: Village Health Worker; WHO: World Health Organization

## Competing interests

The authors declare that they have no competing interests.

## Authors' contributions

LCM, AHB, SEA, and GLD were primarily responsible for the design of the trial and protocol development. AHB and SEA are co-principal investigators of the Projahnmo Chlorhexidine Study. LCM, RS, IM, PJW, SA, MS, SMR, GLD, MRR, and REB are co-investigators. LCM drafted the manuscript. All authors provided critical revision of subsequent drafts and read and approved the final manuscript.

## Pre-publication history

The pre-publication history for this paper can be accessed here:


